# Supplementary material for: Ecological dynamics of Enterobacteriaceae in the human gut microbiome across global populations
Source: Nat Microbiol. 2025 Jan 10;10(2):541–53. doi: 10.1038/s41564-024-01912-6 (PMC11790488; doi:10.1038/s41564-024-01912-6)
Supplement: Supplementary file 1 — Reporting Summary [file 41564_2024_1912_MOESM1_ESM.pdf]

nature portfolio

Corresponding author(s): Alexandre Almeida

Last updated by author(s): Nov 22, 2024

Reporting Summary

Nature Portfolio wishes to improve the reproducibility of the work that we publish. This form provides structure for consistency and transparency in reporting. For further information on Nature Portfolio policies, see our Editorial Policies and the Editorial Policy Checklist.

Statistics

For all statistical analyses, confirm that the following items are present in the figure legend, table legend, main text, or Methods section.

n/a	Confirmed	
☐	☒	The exact sample size (*n*) for each experimental group/condition, given as a discrete number and unit of measurement
☒	☐	A statement on whether measurements were taken from distinct samples or whether the same sample was measured repeatedly
☐	☒	The statistical test(s) used AND whether they are one- or two-sided<br>*Only common tests should be described solely by name; describe more complex techniques in the Methods section.*
☐	☒	A description of all covariates tested
☐	☒	A description of any assumptions or corrections, such as tests of normality and adjustment for multiple comparisons
☐	☒	A full description of the statistical parameters including central tendency (e.g. means) or other basic estimates (e.g. regression coefficient) AND variation (e.g. standard deviation) or associated estimates of uncertainty (e.g. confidence intervals)
☐	☒	For null hypothesis testing, the test statistic (e.g. *F*, *t*, *r*) with confidence intervals, effect sizes, degrees of freedom and *P* value noted<br>*Give P values as exact values whenever suitable.*
☒	☐	For Bayesian analysis, information on the choice of priors and Markov chain Monte Carlo settings
☒	☐	For hierarchical and complex designs, identification of the appropriate level for tests and full reporting of outcomes
☐	☒	Estimates of effect sizes (e.g. Cohen's *d*, Pearson's *r*), indicating how they were calculated

Our web collection on statistics for biologists contains articles on many of the points above.

Software and code

Policy information about availability of computer code

Data collection	fastq-dl v2.0.4. Custom code: https://github.com/alexmsalmeida/metagen-fetch
Data analysis	TrimGalore v0.6.0; BWA MEM v0.7.16a-r1181; CheckM v1.0.11; GUNC v1.0.3; GTDB-Tk v2.3.2; Samtools v1.9; snakemake v7.32.3; ConQuR v1.2.0; Mikropml R package; vegan R package; FastSpar v1.0; ALDEx2 v1.32.0; MaAsLin2 v1.14.1; metaMLST v1.2.3; igraph R package; MEGAHIT v1.2.9; Prodigal v2.6.3; DIAMOND v2.1.8; Panaroo v1.3.3; FastTree v2.1.11; iTOL v6; Prokka v1.14.16; eggNOG-mapper v2.1.3; dbCAN2 v2.0.11; KOFam release 2021-11; gutSMASH v1.0; antiSMASH v6.0.1; CarveMe v1.5.2; PhyloMint v0.1.0; COBRApy v0.29. bowtie2 v2.5.3. Custom code: https://github.com/microfundiv-lab/EnteroEco

For manuscripts utilizing custom algorithms or software that are central to the research but not yet described in published literature, software must be made available to editors and reviewers. We strongly encourage code deposition in a community repository (e.g. GitHub). See the Nature Portfolio guidelines for submitting code & software for further information.

Data

Policy information about availability of data

All manuscripts must include a data availability statement. This statement should provide the following information, where applicable:

- Accession codes, unique identifiers, or web links for publicly available datasets
- A description of any restrictions on data availability
- For clinical datasets or third party data, please ensure that the statement adheres to our policy

All the metagenomic datasets used in this study are publicly available in the European Nucleotide Archive (see Supplementary Table 1 for all associated accession codes). The sequence databases used were retrieved from the Unified Human Gastrointestinal Genome (UHGG) catalog v1.0 and Unified Human Gastrointestinal Protein (UHGP-90) catalog v1.0. Abundance data estimated for the UHGG species and all metagenomic samples here included is available in: https://doi.org/10.6084/m9.figshare.27044341.v1. FASTA files of the BGCs detected with antiSMASH for all co-excluders and co-colonizers can be accessed in: https://doi.org/10.6084/m9.figshare.27044335.v1. Accession code of the human reference genome used for decontamination (GRCh38) is GCA_000001405.15.

Research involving human participants, their data, or biological material

Policy information about studies with human participants or human data. See also policy information about sex, gender (identity/presentation), and sexual orientation and race, ethnicity and racism.

Reporting on sex and gender	N/A
Reporting on race, ethnicity, or other socially relevant groupings	N/A
Population characteristics	N/A
Recruitment	N/A
Ethics oversight	N/A

Note that full information on the approval of the study protocol must also be provided in the manuscript.

Field-specific reporting

Please select the one below that is the best fit for your research. If you are not sure, read the appropriate sections before making your selection.

☒ Life sciences ☐ Behavioural & social sciences ☐ Ecological, evolutionary & environmental sciences

For a reference copy of the document with all sections, see nature.com/documents/nr-reporting-summary-flat.pdf

Life sciences study design

All studies must disclose on these points even when the disclosure is negative.

Sample size	We compiled 12,238 human gut metagenomic samples available in the European Nucleotide Archive (ENA) encompassing 65 different studies from 45 countries (Supplementary Table 1). No sample size calculation was performed. Our analyses revealed that with a subset of the data comprising 5,128 samples from healthy adults we were able to reproduce the results obtained with the full 12,238 dataset, suggesting this sample size is sufficient to identify consistent differences.
Data exclusions	Samples were selected based on the following criteria: 1) containing at least 500,000 paired-end metagenomic reads; 2) with available metadata on health state, age group and country of origin; 3) from individuals with no diagnosed acute infections; and 4) no reported antibiotic usage in the previous month.
Replication	Microbiome signatures linked to Enterobacteriaceae colonization and abundance were confirmed using a subset of samples for healthy adults and using the intersection of three bioinformatics tools (ALDEx2, MaAsLin2 and FastSpar). In addition, machine learning analyses were also confirmed by stratifying samples by continent and performing pairwise cross validation of samples from different continents. We further used study ERP133829 to assess which co-excluder and co-colonizer species were associated with carbapenemase-producing Enterobacteriaceae in particular. Bootstrapping for the FastSpar analysis was repeated 1000 times, whereas machine learning analyses were undertaken with a 5-fold cross-validation repeated 10 times.
Randomization	Samples were classified as Enterobacteriacaea positive or negative based on the detection of any Enterobacteriaceae species using read mapping. Covariates ("Age group", "Continent","Health state", "Read depth" and "Study") were controlled using the generalized and mixed effects models implemented in ALDEx2 and MaAsLin2. In addition, results were confirmed with a subset of samples from healthy adults only. For the machine learning analyses, "Study" was used as a grouping factor to ensure samples from the same study were kept together in either the training or test dataset.
Blinding	Blinding was not relevant to this study, as samples were grouped and categorized based on the detection of Enterobacteriaceae species.

Reporting for specific materials, systems and methods

We require information from authors about some types of materials, experimental systems and methods used in many studies. Here, indicate whether each material, system or method listed is relevant to your study. If you are not sure if a list item applies to your research, read the appropriate section before selecting a response.

Materials & experimental systems

n/a	Involved in the study
☒	☐ Antibodies
☒	☐ Eukaryotic cell lines
☒	☐ Palaeontology and archaeology
☒	☐ Animals and other organisms
☒	☐ Clinical data
☒	☐ Dual use research of concern
☒	☐ Plants

Methods

n/a	Involved in the study
☒	☐ ChIP-seq
☒	☐ Flow cytometry
☒	☐ MRI-based neuroimaging

Plants

Seed stocks	N/A
Novel plant genotypes	N/A
Authentication	N/A
